# SVARAP and aSVARAP: simple tools for quantitative analysis of nucleotide and amino acid variability and primer selection for clinical microbiology

**DOI:** 10.1186/1471-2180-6-21

**Published:** 2006-03-03

**Authors:** Philippe Colson, Catherine Tamalet, Didier Raoult

**Affiliations:** 1 Laboratoire de virologie, fédération hospitalière de bactériologie-virologie clinique et d'hygiène, CHRU Timone, 264 rue Saint-Pierre 13385, Marseille cedex 05, France; 2 Unité des Rickettsies, CNRS UMR 6020 IFR48, faculté de médecine, université de la Méditerranée, 27 boulevard Jean Moulin, 13 385 Marseille cedex 05, France

## Abstract

**Background:**

Simple computerized methods that analyse variability along alignments of nucleotide or amino acid sequences can be very useful in a clinical microbiology laboratory for two main purposes. First, to optimize primer selection, which is critical for the identification of infectious pathogens based on gene sequencing: primers must target conserved nucleotide regions bordering highly variable areas to ensure discrimination of species. Second, it can be of interest to reveal mutations associated with drug resistance of pathogen agents. Our aim was therefore to test easy and cost-free tools (SVARAP and aSVARAP) that require short hands-on work, little expertise, and which allow visual interpretation and statistical analysis of results.

**Results:**

We first tested SVARAP to improve a strategy of identification of streptococci species of the Viridans Group targeting the *groESL *gene. Two regions with <500 nucleotides were identified, one being significantly more discriminant than one of a similar length used in a previous study (mean number of nucleotide differences between species, 113 (range: 12–193) vs. 77 (range: 14–109); *p *< 10^-3^). Secondly, aSVARAP was tested on reverse transcriptase (RT) sequences from 129 HIV-1 clinical strains to identify natural polymorphisms and drug-selected mutations emerging under nucleoside RT inhibitor (NRTI)-selective pressure. It revealed eleven of the 18 RT mutations considered in a reference HIV-1 genotypic NRTI-resistance interpretation algorithm.

**Conclusion:**

SVARAP and aSVARAP are simple, versatile and helpful tools for analysis of sequence variability, and are currently being used in real practice in our clinical microbiology laboratory.

## Background

Sequence variability is a major parameter when designing primers and probes for a new PCR assay, even if various other factors such as string-based alignment scores, melting temperature, primer length and GC contents are also critical [1]. Indeed, nucleotide primers are designed to specifically target a nucleotide region that must be conserved as much as possible in order to ensure their hybridization. Conversely, when nucleotide sequences are used to identify or classify strains, the amplified and then sequenced region has to be divergent enough for discrimination. Variability is also a very informative property of nucleotide and protein sequences. For instance, it may indicate if a region is targeted or not by a given selective pressure or if mutations are occurring under drug-selective pressure.

The analysis of the variability of a genetic or protein region is generally impractical, exacting, and based upon non-objective criteria when performed visually from a multiple sequence alignment. Difficulties are compounded by the length of sequences and their diversity. We therefore developed cost-free tools on Microsoft Excel 2000 software to improve identification and analysis of variable regions in nucleotide and amino acid sequences. These programs, SVARAP (for Sequence VARiability Analysis Program) and aSVARAP (for amino acid Sequence VARiability Analysis Program), use a very simple approach to analyse, reveal, and plot in graphics the variability along multiple nucleotide or amino acid sequences alignments. They combine several advantages: (i) easy handling and interpretation of results, which means quick training of new users, (ii) brief hands-on work (<15 min); (iii) visual interpretation of results that are plotted in graphical windows; (iv) quantification of variability, which allows statistical analysis; (v) versatility, with various targets, such as bacterial or viral genomes, and various purposes, mainly primer or probe design for PCR assays or study of natural and drug-selected polymorphisms. In the present study, in order to illustrate the versatility of our programs, two applications for clinical microbiology were tested: firstly, to design primers for sequence amplification and identification in streptococci, and secondly, to identify natural polymorphisms and drug-selected mutations in HIV-1.

## Implementation

SVARAP user manual is available on the World Wide Web [2]. SVARAP can simultaneously process and analyse sets of up to 100 sequences with a maximal length of 4,000 nucleotides for each sequence. All sequences of the studied set of sequences are aligned with ClustalX, version 1.83 [3]. SVARAP uses an alignment in GDE format (Genetic Data Environment) generated by ClustalX. Aligned sequences are copied, then pasted into a cell of the main page of our Microsoft Excel file and automatically processed. Each nucleotide for each sequence is automatically assigned to a different cell in order to align nucleotides corresponding to a given position in the alignment in successive lines of the same column. The SVARAP calculates for each nucleotide site in the alignment the consensus nucleotide (defined as the most common nucleotide at a site in the studied set of sequences), the number of different nucleotides, the absolute number of each of the four nucleotides (G, A, C, and T) or the number of deletions or insertions, and their frequencies (in percentages). We tabulated site variability as the proportion of sequences that differ from the consensus at a given site. Variability is calculated as follows: 100 – (maximum frequency for each of the four nucleotides at a given position). The data are also processed to calculate median, mean, highest and lowest variability for a non-sliding window of 50 nucleotides, with standard deviations. Moreover, SVARAP calculates along the whole alignment the mean variability for a sliding window of 25 nucleotides. This length was chosen because it approximately corresponds to the length of a primer. Thus, simultaneous site-by-site analysis and nucleotide window analysis can be performed to reveal the regions with particular patterns of variability. All of these data are available in different sheets in tables or are plotted in graphical windows to allow visual interpretation. All tables or figures are printable. Hypertext links allow access to these tables or plots, and easy return to the home page. The quantitative results obtained from SVARAP can be copied for further comparison of data obtained from several sets of sequences, or graphical representation of the sequence variability for longer regions (>4,000 nucleotides) than initially proposed. An example of the use of SVARAP is available on the World Wide Web [4].

The aSVARAP is an *in-house *program derived from SVARAP and dedicated to amino acid sequences, the principle and use being the same as for SVARAP. We first generate with ClustalX v.1.83 an alignment from a set of up to 100 sequences with a maximal length of 1,000 amino acids for each sequence. Then, our program calculates the proportion of sequences harboring an amino acid at a given position that is not the most frequently found in the studied set of sequences.

## Results

### Test of SVARAP on VGS *groESL *sequences

SVARAP was used on Viridans Group streptococci (VGS) *groESL *sequences. VGS are examples of bacteria for which it is important to accurately identify the species. Indeed, they are capable of causing many serious infections such as subacute infective endocarditis, and clinical significance and antimicrobial susceptibility may differ between species [5,6]. Teng et al. studied ten species of VGS [7]. They previously amplified and sequenced VGS *groESL *gene regions using degenerated primers and LA-PCR. This strategy failed to amplify six out of ten VGS species, and six other primers were thus used to target the *groES *and/or the *groEL *genes of VGS. Four primers were used to amplify and sequence the *GroES *gene and four other primers were used for the *GroEL *gene. They finally determined VGS *groESL *full-length sequences and a strategy for VGS identification using *groES*, *groEL *and the intergenic region (spacer) between the *groES *and *groEL *genes. Then, they studied nucleotide sequence similarity scores between VGS species in the *GroES *and *GroEL *genes. We investigated if better strategies to amplify and differentiate VGS could be obtained using SVARAP. We used all of the sequences determined by Teng et al. and the *groESL *sequence from *Streptococcus pneumoniae*, which was also analysed by these authors [7]. Sequences were obtained from the GenBank sequence database. The accession numbers were as follows: *Streptococcus anginosus*, AF378195; *Streptococcus constellatus*, AF378196; *Streptococcus intermedius*, AF389515; *Streptococcus sanguis*, AF378197; *Streptococcus gordonii*, AF338228; *Streptococcus oralis*, AY38047; *Streptococcus mitis*, AF417589; *Streptococcus bovis*, AF389514; *Streptococcus mutans*, AF389516; *Streptococcus salivarius*, AF389517; *Streptococcus pneumoniae*, AF117741. Sequences comprised between 1,963 and 3,666 nucleotides and were processed using ClustalX and SVARAP as described above. Then, regions were chosen for their variability features after visual analysis of the graphics produced by SVARAP. Nucleotide variability for both windows of 25 and 50 nucleotides and site by site were considered. A more precise analysis was also possible to be performed using numerical data available in tables.

**Figure 1:**
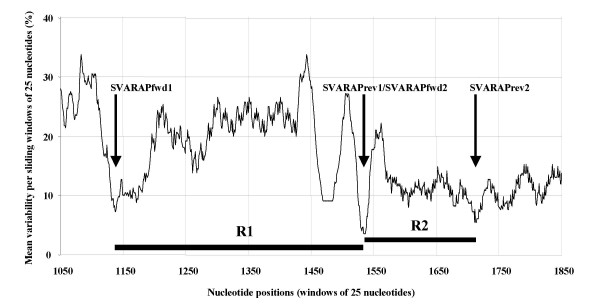
**Nucleotide variability per sliding window of 25 nucleotides in *groESL *sequences of eleven VGS reference strains**. Arrows indicate positions chosen using SVARAP for primer sequence design. R1 and R2 are the two discriminant regions bordered by conserved areas revealed using SVARAP. SVARAPfwd1 and SVARAPrev1 are the primers designed for R1 amplification and sequencing; SVARAPfwd2 and SVARAPrev2 are the primers designed for R2 amplification and sequencing.

When starting from ClustalX *GroESL *sequence alignment, less than fifteen minutes were necessary to obtain the results provided by SVARAP – this time mostly consisted in formatting the GDE sequence alignment generated by ClustalX to make it fit with SVARAP processing. The interpretation of SVARAP results was then performed in only fifteen more minutes. Three interesting sites for primer hybridization were clearly revealed and easily visualized on the plot representing the mean variability for sliding windows of 25 nucleotides (Figure 1). They were the most conserved with mean variability ranging from 4 to 7%. Moreover, they bordered two very polymorphic regions with mean variability reaching 27% for the first nucleotide region (R1) and 22% for the second nucleotide region (R2). R1 (up to 371 nucleotides) partially includes the *groES *gene in its 3' region and the intergenic region between *groES *and *groEL*. R2 (159 nucleotides) is located in the *groEL *5' region. SVARAP site-by-site analysis showed precisely the location of variable positions in the putative primer binding sites, their level of variability, as well as the number and nature of nucleotides harbored. The sequences of the three primers were as follows: SVARAPfwd1: 5'-AAACCMTTRGGNGAYCGWRTSST; SVARAPrev1/SVARAPfwd2: 5'-TGKCAAAAGAHATTAAATTTTCA; SVARAPrev2: 5'-GAAGAYCAYTTTGAAAAYATGGG.

In the *groEL *gene, the primer binding sites underlined by SVARAP for amplification and sequencing of VGS are less degenerated than those targeted by Teng et al. despite having been designed to hybridize with all eleven VGS species [7]. The R2 forward primer SVARAPfwd2 has two (8.6%) degenerated positions out of 23, involving two and three different nucleotides. The R2 reverse primer SVARAPrev2 carries three (13.0%) degenerated sites out of 23, involving two different nucleotides each. In comparison, the two primers used by Teng et al. for clinical isolates and in a first step for VGS reference strains carried six (23.1%) degenerated positions out of 26 for the forward primer, all corresponding to N, and eight (30.8%) out of 26 for the reverse primer (all but two corresponding to N) [7]; moreover, they failed to amplify six out of the ten VGS reference strains and two others primers were used to obtain the full length or near-full length sequence of the *groEL *gene; they included three (14.3%) and four (16.0%) degenerated positions. Overall, in the *groEL *gene, the number of degenerated sites was significantly lower for R2 primers pair compared with the one used by Teng et al. (5 vs. 14; p = 0.046). For the *groES *gene, four primers were designed by Teng et al.. The sequences of two were available and comprised three (14.3%) degenerated positions for the reverse primer and no degenerated site for the forward primer. Two other primers were designed to amplify the entire *groES *sequence and the *groES*-*groEL *spacer region. The R1 forward primer SVARAPfwd1 carried eight (34.8%) degenerated positions out of 23. However, in seven cases they involved only two nucleotides. Of note, if the two sequences of *S. mutans *and *S. salivarius *were excluded, the number of degenerated positions would decrease to three (13.0%). The R1 reverse primer SVARAPrev1 corresponds to primer SVARAPfwd2 which comprises two (8.6%) degenerated positions. Finally, SVARAP strategy would use two primers, and could target either the *groES *or the *groEL *region.

**Figure 2:**
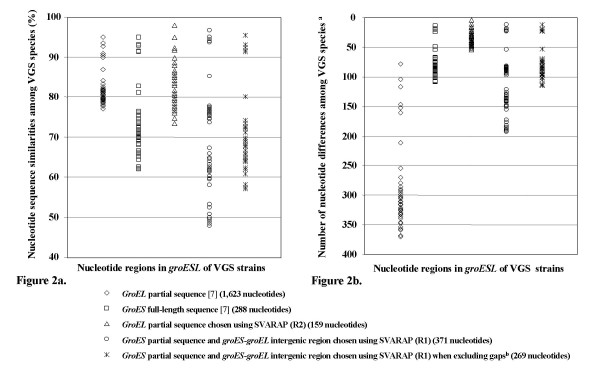
**Nucleotide sequence similarities (figure 2a) and numbers of nucleotide differences (figure 2b) among VGS species when analyzing *groEL *and *groES *sequences from the study by Teng et al [7], or regions R1 and R2 chosen using SVARAP to discriminate between the different VGS species**. ^a ^For sequences obtained by Teng et al., the numbers of nucleotide differences among VGS species were calculated based on the nucleotide sequence similarities and the sequence lengths published [7]. ^b ^Partial *groES*-including sequences chosen using SVARAP (R1) were analysed to calculate sequence similarities considering or not considering positions in the alignment where at least one sequence does not harbor a nucleotide (gaps).

On the other hand, based on nucleotide sequence similarity scores between the eleven reference strains of VGS described above, R1 was more polymorphic than the *groEL *region analysed by Teng et al. (mean nucleotide sequences similarity scores between species of 69.7% (range: 48.0–96.8) vs. 81.7% (range: 77.2–95.2); *p *< 10^-3^), whereas it showed a tendency for higher variability than *groES *full-length sequences (73.3% (range: 62.1–95.1); *p *= 0.083) (Figure 2a). Also, the proportion of nucleotide sequence similarity scores between VGS species that were <80% was significantly higher in R1 than in Teng et al's *groEL *region (87.3% vs. 38.2%; *p *< 10^-3^). When analyzing the mean number of inter-species nucleotide differences, R1 was significantly more discriminant than *groES *full-length region used by Teng et al. (113 nucleotide differences (range: 12–193) vs. 77 (range: 14–109); *p *< 10^-3^) (figure 2b). Moreover, R1 includes *groES *and *groEL *intergenic spacer that vary according to the species in sequence and in length, ranging from 15 to 111 nucleotides [7]. R2 is also a very interesting region for differentiation of VGS species. Very conserved primer binding sites border it and it is a very short sequence. Fifty-four (98.2%) of the sequence comparisons of VGS species retrieved more than 10 nucleotide differences, and 51 (92.7%) retrieved more than 20 differences. The *GroEL *region studied by Teng et al. displayed significantly more nucleotide differences among VGS species than R2 and R1 (*p *< 10^-3^) (Figure 2b), but this is due to its length that overruns 1,500 nucleotides. In fact, this is a strong drawback because, at the present time, routine sequencing assays produce, in a single run, sequences of a maximum length of 600 nucleotides. Conversely, R1 and R2 (up to 417 nucleotides for R1 and 205 nucleotides for R2, including primers) would be easily sequenced. With regard to R1 and R2 phylogenetic analysis, species within a VGS group were found to be highly related, as found by Teng et al.: for example, *S. anginosus*, *S. constellatus *and *S. intermedius *were highly related (bootstrap values of 97 and 93 for R1 and R2, respectively), as were *S. mitis*, *S. oralis *and *S. oralis *(bootstrap values of 97 and 100 for R1 and R2, respectively).

### Test of aSVARAP on HIV-1 RT sequences

The version of SVARAP dedicated to analysis of variability in amino acid sequences, called "aSVARAP", was used in the setting of HIV-1 infection. Genotypic testing of resistance to antiretroviral compounds is recommended in clinical practice to guide the therapeutic management of HIV-1-infected patients [8]. This corresponds to the amplification and sequencing of the reverse transcriptase (RT) and protease coding regions in HIV-1 genome. RT is one of the two enzymes targeted by antiretrovirals (ARV) available to date [9]. Knowledge of amino acid changes, especially under the selective pressure of antiretroviral therapies, usually requires large-scale studies and clinical, virological, and immunological results together with HIV genotypic and phenotypic susceptibility data. We sought to evaluate if aSVARAP was a reliable tool to characterize the polymorphism of HIV-1 RT in the absence of drug and to identify mutations emerging under NRTI-selective pressure.

**Figure 3:**
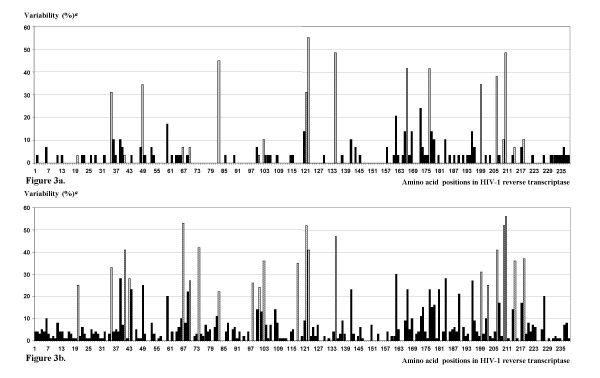
**Amino acid variability at each site of HIV-1 reverse transcriptase for untreated individuals (n = 29, figure 3a) and patients treated with nucleoside reverse transcriptase inhibitor-containing regimen (n = 100, figure 3b)**. Gray rods indicate positions where variability exceeded 30%. Hachured rods indicate positions where differences between variability in treated and untreated patients exceeded 20%. ^a ^Variability was considered as the percentage of sequences that harbored (at a given position) an amino acid that was not the most frequently found in the studied set of sequences.

HIV-1 RT coding regions from 29 HIV-1-infected patients who had never received antiretroviral therapy and 100 HIV-1-infected patients who had been exposed to various combinations of nucleoside reverse transcriptase inhibitors (NRTI) were studied. The treated patients who were selected were the first 100 in alphabetic order for whom a genotypic testing of resistance to antiretrovirals was prescribed during the previous year. The untreated patients who were selected had been recently HIV-diagnosed. All sequences were obtained from our HIV sequence database [10]. Sequences comprised 717 nucleotides and were processed using ClustalX and aSVARAP as described above for nucleotide sequences and SVARAP. We studied the 239 first codons of the HIV-1 RT gene from 129 HIV-1-infected patients who were exposed or not to nucleoside reverse transcriptase inhibitors (NRTI) (Figures 3). The aSVARAP firstly helped to reveal that HIV-1 strains harbored areas of natural polymorphism. We observed that 99 (41.4%) nucleotide positions were variable in the absence of NRTI-selective pressure (Figure 3a). Codons 35, 49, 83, 122, 123, 135, 167, 177, 200, 207 and 211 exhibited a variability >30% in the absence of antiretroviral therapy. In contrast, three regions from codons 71 to 82, 91 to 99 and 147 to 157 were fully conserved in the absence of drug-selective pressure. These conserved regions could be crucial for RT functionality and thus appropriate targets for ARV. Secondly, when comparing variability in the presence or in the absence of reverse transcriptase inhibitors-selective pressure (figures 3a and 3b), we observed differences exceeding 10% at 34 (14.2%) positions. Of the 16 positions where this difference was >20%, ten harbored previously well-defined drug-resistance mutations (at positions 41, 67, 70, 74, 101, 103, 118, 210, 215, 219) [8] – two of which occurred in the presence of non nucleoside reverse transcriptase inhibitors (NNRTI) which were poorly represented in the drug combination regimen of the 100 selected patients. All five positions (41, 67, 74, 118, 210) where the difference between amino acid variability of HIV-1 RT from treated and untreated patients exceeded 30% were associated with HIV-1 NRTI-resistance mutations. Moreover, we considered positions where amino acid variability was significantly higher in RTI-treated versus naive patients (*p *< 0.05). Eleven positions (41, 44, 67, 69, 70, 74, 118, 184, 210, 215, 219) harbored previously well-defined NRTI-resistance mutations, and four positions (101, 103, 181, 190) harbored well-defined NNRTI-resistance mutations. Of seven other positions, four (20, 43, 98 and 203) were highly conserved (variability <5%) in HIV-1 strains from untreated individuals, and should be further analysed to verify if they are not involved in drug-resistance. Seven HIV-1 RT positions (62, 65, 75, 77, 115, 116, 151) previously described as harboring NRTI-resistance mutations were not revealed by aSVARAP as being more variable in treated patients than in untreated individuals. This could be related to the low frequencies of mutations observed at these sites in clinical practice, and indeed, they all displayed low variability between 2% and 7% under NRTI-selective pressure.

## Discussion

SVARAP and aSVARAP, two *in-house *programs, were used in our clinical microbiology laboratory for two applications related to routine and clinical research assays. The aim was to test their facility, rapidity and usefulness for analysis of nucleotide or amino acid sequence variability.

SVARAP revealed two nucleotide regions R1 and R2 to amplify all VGS and to identify VGS species. A single primers pair could then be used for each of those two regions. R1 is globally more discriminant with VGS species than *groES *full-length sequences, and tend to be more discriminant than the large *groEL *region previously analysed by Teng et al. [7]. R2 is less variable but is also discriminant, and it has more conserved primer-binding sites. Indeed, R2 primers targeting the *groEL *gene are significantly less degenerated than those used by Teng et al. [7]. Moreover, R1 and R2 shortness (<500 nucleotides) means time and cost-saving sequencing procedures. Each could be used separately for PCR amplification and species differentiation. ASVARAP was helpful to characterize the polymorphism of HIV-1 RT in the absence of drugs and to identify mutations emerging under NRTI-selective pressure, even if only 129 out of 7,000 RT sequences were processed from our clinical database. Major data were obtained easily and quickly without performing a large-scale study, and aSVARAP revealed 11 of the 18 RT mutations that are considered in the HIV-1 genotypic NRTI-resistance interpretation algorithm of the International AIDS Society [8]. The seven mutations that were not detected occur with very low frequency in clinical practice.

Several programs do exist that analyse variability in nucleotide and/or amino acid sequences. For instance, SWAN performs very sophisticated analysis by estimating the variability in each column of an alignment of nucleotide sequences as an entropy function of the nucleotide variation observed at this site [11]. It permits calculation and representation of the first and second derivatives of variability, providing information about the regularity of sequence variation in genomes. Several other programs are available commercially or freely on the World Wide Web to design primers for PCR assays. For instance, Oligo Primer Analysis Software (Molecular Biology Insights, Inc), which needs to be purchased, takes into account various other critical parameters than sequence variability, including melting temperature, primer length, GC contents, or the formation of secondary structures, as hairpins and self and cross dimers [1,12]. Nevertheless, extensive expertise is required to take advantage of all these parameters. In contrast, SVARAP uses a very simple approach for basic analysis of genetic variability. It reveals the most conserved areas whatever the degree of divergence between the studied sequences, and provides the frequencies of all different nucleotides at degenerated sites in designed primers. Moreover, the variability of the amplified regions can also be considered. Thus, SVARAP allows to seek at a glance for regions with high variability flanked by conserved areas for sequence-based differentiation and identification of various infectious pathogens.

Regarding programs that analyse variability in amino acid sequences, the detection of mutations emerging under drug selective pressure in HIV-1, which was performed using aSVARAP in the present study, can be done using the Stanford Drug Resistance Database Program (SDRDP). SDRDP is in free access on the World Wide Web [13], and is able to provide results based on a large sequence database in a few seconds [14]. However, this program is dedicated to the analysis of HIV-1 protease and reverse transcriptase sequences, whereas aSVARAP is a versatile tool that permits to analyse any set of sequences consisting in different regions of different infectious pathogens. Furthermore, aSVARAP is able to compare sets of sequences in the absence or in the presence of several different selective pressures. AL2CO is another program that analyses amino acid variability [15]. It can help to predict functionnaly and/or structurally important sites, based on the principle that these latter tend to be more conserved. It attributes to each aligned amino acid position in a protein sequence alignment a conservation index that is defined using three conceptually different approaches: entropy-based, variance-based and matrix score-based. Although aSVARAP performs less sophisticated analysis of amino acid variability, it provides easy interpretable results including the level of variability and the frequencies of the different amino acids at each position in a multiple sequence alignment.

It cannot be ruled out that, in biology, *in silico *studies are not fully superposable to real practice. In fact, several users from different departments of our clinical microbiology laboratory have tested SVARAP or aSVARAP successfully. Adekambi et al. used SVARAP in the setting of non-pigmented and late-pigmenting rapidly-growing mycobacteria (RGM), which are increasingly isolated in clinical microbiology laboratories [16]. *16S rRNA *gene sequence analysis underestimates RGM diversity and does not distinguish between all taxa. Adekambi et al. therefore determined the complete nucleotide sequence of the *rpoB *gene, which encodes the bacterial beta subunit of the RNA polymerase, for 20 RGM type strains and they focused, after using SVARAP, on a 723-bp variable region exhibiting 83.9 to 97% interspecies similarity and 0 to 1.7% intraspecies divergence. The latter was further applied to the identification of 63 RGM clinical isolates: 59 (94%) exhibited <2% partial *rpo*B gene sequence divergence with one of 20 species under study and were regarded as correctly identified at the species level. In another study, Khamis et al. investigated the usefulness of *rpoB *sequencing for differentiation and identification of bacteria belonging to the genera *Afipia *and *Bosea *which are amoeba-resisting bacteria recently reported to colonize hospital water supplies [17,18]. The major drawback of *rpoB *sequencing is that the length of the gene (>4,000 bp) does not allow routine molecular identification or detection in clinical samples. Conversely, SVARAP allowed the design of universal primers for amplification and sequencing of a 740- to 752-bp fragment containing a hypervariable region of 408 to 420 bp for identification of all species tested in the phylum. The version of SVARAP dedicated to analysis of variability in amino acid sequences, called "aSVARAP", was used in the setting of infection with human immunodeficiency virus type 2 (HIV-2) [19]. Because of its limited worldwide spread, little data is available concerning the susceptibility of human immunodeficiency virus type 2 (HIV-2) to protease inhibitors (PI) and the potential emergence of resistance [20]. Therefore, 56 HIV-2 protease sequences from 21 untreated or NRTI-treated HIV-2-infected patients were analysed in our laboratory to identify mutations emerging under PI-selective pressure [19]. We were confronted with the difficulty of revealing drug-resistance mutations from a limited number of sequences based on objective and statistical criteria. Finally, mutations were considered to be selected under PI-containing antiretroviral regimen based on the presence of at least the following criteria: (i) a significantly higher amino acid variability at a given position in PI-treated versus naive patients; (ii) a statistically significant difference between the frequency of amino acids at a given position in naive versus PI-treated patients. A significantly higher amino acid variability was found at positions 7, 62, 71, 90 and 99 in PI-treated patients than in treatment-naive patients (*p *< 0.05). Moreover, the frequencies of amino acids 46I, 62A or T, 71I, 90M and 99F, automatically calculated by SVARAP, were significantly higher in the PI-treated patients than in the untreated patients (*p *< 0.05). These drug-selected mutations were in line with longitudinal studies of PI-treated patients in therapeutic failure.

## Conclusion

SVARAP and aSVARAP are very simple, versatile and helpful tools for analysis of genetic or protein variability. They do not require time-consuming or extensive expertise for manipulation or interpretation. At present, they are frequently used in our clinical microbiology laboratory by different users for analysis of rapidly growing sequence databases. Users mostly seek regions with high variability flanked by conserved areas, optimal for PCR and sequencing, as well as natural polymorphism or drug-selected mutations. However, SVARAP could be applied to various other purposes. For instance, it could be used in multilocus sequence typing (MLST), an emerging typing method based on sequence comparisons of multiple loci [21].

## Availability and requirements

SVARAP and aSVARAP are free and downloadable on the World Wide Web [22]. They are Microsoft Excel files and only Microsoft Excel 2000 software is required for their use. User manual is available on the World Wide Web [2]. ClustalX v 1.83 is free and downloadable [23].

## List of abbreviations used

IUPAC ambiguity codes have been used in the primer sequences [24].

## Authors' contributions

PC conceived the programs, carried out in silico testings and drafted the manuscript. CT and DR participated in the design and coordination of the study, and helped to draft the manuscript. All authors read and approved the final manuscript.
